# Long-term follow-up of ibrutinib monotherapy in treatment-naive patients with Waldenstrom macroglobulinemia

**DOI:** 10.1038/s41375-021-01417-9

**Published:** 2021-09-16

**Authors:** Jorge J. Castillo, Kirsten Meid, Joshua N. Gustine, Carly Leventoff, Timothy White, Catherine A. Flynn, Shayna Sarosiek, Maria G. Demos, Maria L. Guerrera, Amanda Kofides, Xia Liu, Manit Munshi, Nicholas Tsakmaklis, Lian Xu, Guang Yang, Andrew R. Branagan, Elizabeth O’Donnell, Noopur Raje, Andrew J. Yee, Christopher J. Patterson, Zachary R. Hunter, Steven P. Treon

**Affiliations:** 1 Bing Center for Waldenström Macroglobulinemia, Dana-Farber Cancer Institute, Boston, MA USA; 2 Department of Medicine, Harvard Medical School, Boston, MA USA; 3 Department of Medicine, Boston University School of Medicine, Boston, MA USA; 4 Center for Multiple Myeloma, Massachusetts General Hospital, Boston, MA USA

**Keywords:** Targeted therapies, B-cell lymphoma

## Abstract

Herein, we present the final report of a single-center, prospective phase II study evaluating ibrutinib 420 mg once daily in 30 treatment-naive patients with Waldenstrom macroglobulinemia (WM). The present study is registered with ClinicalTrials.Gov (NCT02604511). With a median follow-up of 50 months, the overall, major, and VGPR response rates were 100%, 87%, and 30%. The VGPR rate was numerically but not significantly lower in patients with than without *CXCR4* mutations (14% vs. 44%; *p* = 0.09). The median time to a minor response was 0.9 months, and to a major response was 1.9 months, though were longer in those with mutated *CXCR4* at 1.7 months (*p* = 0.07) and 7.3 months (*p* = 0.01). Six patients had disease progression. The median progression-free survival (PFS) was not reached, and the 4-year PFS rate was 76%. There was also a non-significant lower 4-year PFS rate in patients with than without *CXCR4* mutations (59% vs. 92%; *p* = 0.06). The most common treatment-related adverse events were fatigue, upper respiratory infection, and hematoma. Atrial fibrillation occurred in 20% of patients. Ibrutinib monotherapy induced durable responses in treatment-naive patients with WM. *CXCR4* mutations impacted VGPR attainment, time to major response, and 4-year PFS rate.

## Introduction

The genomic landscape of Waldenstrom macroglobulinemia (WM) is composed of recurrent mutations in the *MYD88* and *CXCR4* genes, which can be detected in 90–95% and 30–40% of patients with WM, respectively [1, 2]. The activation of the Bruton tyrosine kinase (BTK) pathway plays an essential role in cell survival in *MYD88*-mutated WM cells [3]. In preclinical studies, BTK inhibition induced cell death in WM cell lines and primary WM cells, while *CXCR4* mutations promoted resistance by overexpression of AKT and ERK [3, 4].

In 2015, the United States Food & Drug Administration approved the oral BTK inhibitor ibrutinib for the treatment of patients with symptomatic WM, based on the results of an investigator-initiated multicenter phase II study in 63 previously treated patients in which ibrutinib monotherapy induced an overall response rate (ORR) of 91% and a major response rate of 71% [5]. The long-term data from this study were recently published and reported a 5-year progression-free survival (PFS) rate of 54% [6]. *CXCR4* mutations were associated with a longer time to response, lower rates of major response and very good partial response (VGPR), and a shorter PFS.

We previously reported early data on ibrutinib as primary therapy in patients with WM [7]. This report presents the long-term follow-up from this investigator-initiated, single-center, phase II study evaluating ibrutinib monotherapy in treatment-naive patients with WM.

## Methods

### Study design and oversight

This study was an investigator-initiated, open-label, prospective trial of single-agent ibrutinib in treatment-naive WM patients. Enrollment began January 26, 2016, and closed December 28, 2016. The data cutoff was March 16, 2021. The study was approved by our institutional review board, and all patients provided written informed consent for participation. Pharmacyclics provided research funding and study drug. The trial was registered under ClinicalTrials.Gov ID NCT02604511.

### Study patients

**Fig. 1:**
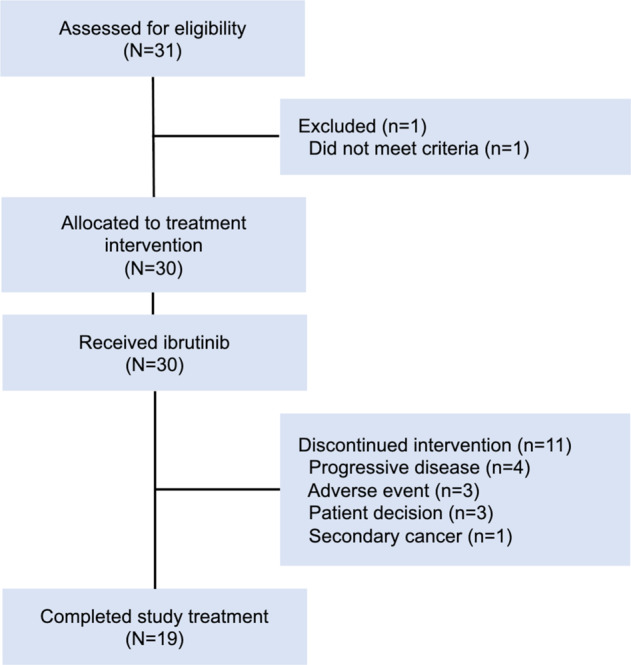
CONSORT diagram.

Patients received ibrutinib on study for 48 months with commercial drug supply after that. All patients met diagnostic criteria for WM and met criteria for treatment initiation based on symptomatic disease [8, 9]. Eligibility criteria included platelet count ≥50 × 10^9^/L, hemoglobin level ≥8 g/dl, absolute neutrophil count (ANC) ≥1.0 × 10^9^/L, serum creatinine level <2 mg/dl, total bilirubin level <1.5 mg/dl (or < 2.0 mg/dl, if attributable to tumor), serum AST and ALT levels <2.5 times the upper limit of normal, and Eastern Cooperative Oncology Group performance status <2. Patients with central nervous system involvement, with active infection with HIV, hepatitis B, or hepatitis C, with clinically significant cardiovascular disease, on warfarin, or on medications that could prolong QT interval were excluded. A CONSORT diagram for patient enrollment and disposition is shown in Fig. 1.

### Study treatment

Intended therapy consisted of oral ibrutinib at 420 mg orally once daily until disease progression or unacceptable toxicity. Ibrutinib was held for ANC < 0.5 × 10^9^/L, platelets <25 × 10^9^/L or <50 × 10^9^/L with bleeding, grade ≥3 nausea, vomiting or diarrhea, or grade ≥3 nonhematological toxicities. Filgrastim injections and transfusion support were permitted. Full-dose retreatment was permitted after toxicity recovery from first drug hold, but thereafter, reduction to 280 mg (dose level −1), then 140 mg (dose level −2), then discontinuation was required with subsequent events. Temporary drug holds for 3–7 days before and after invasive procedures were recommended to minimize bleeding risk.

### Assessments

Responses were assessed using modified criteria from the 6th International Workshop on WM [10]. A decrease of 25–49%, 50–89%, and ≥90% in serum IgM levels (or normalization of IgM levels with persistent monoclonal protein on serum immunofixation electrophoresis) denoted minor response, partial response (PR), and VGPR, respectively. Improvement in extramedullary disease was required for VGPR but not for minor responses or PR attainment. Normalization of serum IgM level, without detectable monoclonal IgM paraprotein, BM disease involvement, or pathological adenopathy, or splenomegaly were required for complete response (CR). ORR included minor response or better, and major response rate included PR or better. PFS was defined as the time between initiation of therapy and date of disease progression, death, or last follow-up. The primary objective was to determine the ORR to ibrutinib monotherapy. Secondary objectives included determination of major response rate, PFS, and drug safety. A serum IgM level increase during temporary holds was not considered disease progression. Bone marrow biopsies and computed tomography scans (if extramedullary disease was present at baseline) were repeated at cycles 6, 12, 24, and as needed thereafter for disease assessment. Adverse events were graded according to the National Cancer Institute Common Terminology Criteria for Adverse Events, version 4.03.

An allele-specific polymerase chain reaction (PCR) assay was used to detect *MYD88 L265P* mutation [2]. *CXCR4* mutational status was determined by Sanger sequencing for frameshift mutations and allele-specific PCR for nonsense mutations [1]. These tests were performed on CD19-selected bone marrow lymphoplasmacytic cells.

We calculated the number of doses of study therapy expected to be taken by each patient and the number of doses missed or temporarily held for procedures or toxicity during study therapy. We subtracted the number of missed or held doses from the expected number of doses and divided the result by the total expected number of doses to estimate ibrutinib dose intensity.

### Statistical analysis

A sample size of 30 participants was estimated to detect a difference between a null ORR of ≤40% and an alternative ORR of ≥70% with a power of 90% and a two-sided alpha of 0.05, based on previously reported efficacy of ibrutinib monotherapy [5, 11]. Data were categorized as follows: age (≤65 or >65 years), sex (male or female), serum IgM level (<4000 or ≥4000 mg/dl), hemoglobin level (≤11.5 or >11.5 g/dl), platelet count (≤100 or >100 × 10^9^/L), serum beta-2-microglobulin level (≤3 or >3 mg/dl), bone marrow involvement (<60% or ≥60%), IPSSWM risk category (low, intermediate or high), any lymphadenopathy ≥1.5 cm (yes or no), splenomegaly ≥15 cm (yes or no), *CXCR4* mutational status (wild-type or mutated), and time from diagnosis to ibrutinib initiation (<12 months or ≥12 months). All these factors were evaluated in categorical response, time to response, and survival analyses.

The Fisher’s exact test and the rank-sum test were used for comparisons between groups for categorical and continuous variables, respectively. The Kaplan–Meier method was used for time-to-response assessments, and the log-rank test for comparisons between groups. Univariate logistic regression models were fitted to estimate the odds ratio (OR) of attaining a major response and a VGPR with 95% confidence intervals (CI). Univariate Cox proportional-hazard regression models were fitted to estimate the hazard ratio (HR) of progression or death with 95% CI. P values <0.05 indicate statistical significance. Statistical analyses and graphs were obtained using STATA 17 (College Station, TX, USA).

## Results

### Patients’ baseline characteristics

Thirty-one patients were enrolled in this study. One patient was ineligible after a lymph node biopsy showed transformation to diffuse large B-cell lymphoma before protocol therapy was started. The *MYD88 L265P* mutation was detected in all patients. *CXCR4* mutations were detected in 14 patients (47%), of which 13 had nonsense (11 *CXCR4 S338X*, 1 *CXCR4 R334X*, and 1 *CXCR4 S324F*), and one had a frameshift mutation (*CXCR4 T318fs*). The clinical characteristics of the participants according to *CXCR4* mutational status are shown in Table 1. Patients with *CXCR4* mutations had lower rates of lymphadenopathy than patients without *CXCR4* mutations (14% and 50%; p = 0.04). There was a trend toward a lower median serum β2-microglobulin level in patients with than without *CXCR4* mutations (3.4 and 4.2 mg/L; *p* = 0.07). There were no other detectable differences between patients based on *CXCR4* mutational status.

**Table 1:** Patients’ characteristics according to *CXCR4* mutational status.

Characteristic	All participants (*n* = 30)	*CXCR4 WT* (*n* = 16)	*CXCR4 MUT* (*n* = 14)	*p*
Age				
At WM diagnosis, median (range)—years	64 (43–81)	63 (43–81)	64 (43–71)	0.93
At ibrutinib initiation, median (range)—years	67 (43–83)	67 (43–83)	67 (43–75)	0.87
>65 years at ibrutinib initiation (%)	19 (63)	10 (63%)	9 (64%)	1.00
Sex				
Male (%)	23 (77)	12 (75%)	11 (79%)	1.00
Female (%)	7 (23)	4 (25%)	3 (21%)	
Serum IgM level				
Median (range)—mg/dl	4370 (844–10,321)	3928 (858–10,321)	5294 (844–7450)	0.58
≥4000 mg/dl (%)	16 (53)	8 (50%)	8 (57%)	0.73
Hemoglobin level				
Median (range)—g/dl	10.3 (7.5–14.4)	10.1 (8.6–14.4)	10.6 (7.5–13.5)	0.60
≤11.5 g/dl (%)	23 (77)	13 (81%)	10 (71%)	0.42
Platelet count				
Median (range)—×10^9^/L	247 (59–491)	288 (129–418)	199 (59–491)	0.13
≤100 × 10^9^/L (%)	2 (7)	0 (0%)	2 (14%)	0.21
Serum beta-2-microglobulin level				
Median (range)—g/dl	3.8 (2–7.6)	4.2 (2.3–6.9)	3.4 (2–7.6)	0.07
>3 g/dl (%)	22 (73)	13 (81%)	9 (64%)	0.42
IPSSWM score				
Low risk (%)	5 (17)	3 (19%)	2 (14%)	0.39
Intermediate risk (%)	11 (37)	4 (25%)	7 (50%)	
High risk (%)	14 (47)	9 (56%)	5 (36%)	
Bone marrow involvement				
Median (range) - %	65 (5–95)	60 (5–95)	70 (10–90)	0.57
≥60% (%)	22 (73)	10 (63%)	12 (86%)	0.23
Extramedullary disease				
Adenopathy ≥1.5 cm (%)	10 (33)	8 (50%)	2 (14%)	0.04
Splenomegaly ≥15 cm (%)	5 (17)	4 (25%)	1 (7%)	0.34
Time from WM diagnosis to ibrutinib				
<12 months (%)	16 (53)	10 (63%)	6 (43%)	0.46
≥12 months (%)	14 (47)	6 (38%)	8 (57%)	

*WM* Waldenstrom macroglobulinemia, *IPSSWM* International Prognostic Scoring System for WM, *WT* wildtype, *MUT* mutated.

Indications to treat included constitutional symptoms (*n* = 21; 70%), symptomatic anemia (*n* = 19; 63%), symptomatic hyperviscosity (*n* = 6; 20%), symptomatic extramedullary disease (*n* = 2; 7%), progressive peripheral neuropathy (*n* = 2; 7%), symptomatic cryoglobulinemia (*n* = 1; 3%), and symptomatic autoimmune hemolytic anemia (*n* = 1; 3%). Nineteen patients (63%) had more than one criterion to treat. Three patients had acquired von Willebrand disease (aVWD) with Willebrand antigen, ristocetin cofactor, and factor VIII levels of <50%. All the patients with aVWD harbored a *CXCR4* mutation and had a serum IgM level >6000 mg/dl at baseline.

### Response to therapy

**Fig. 2:**
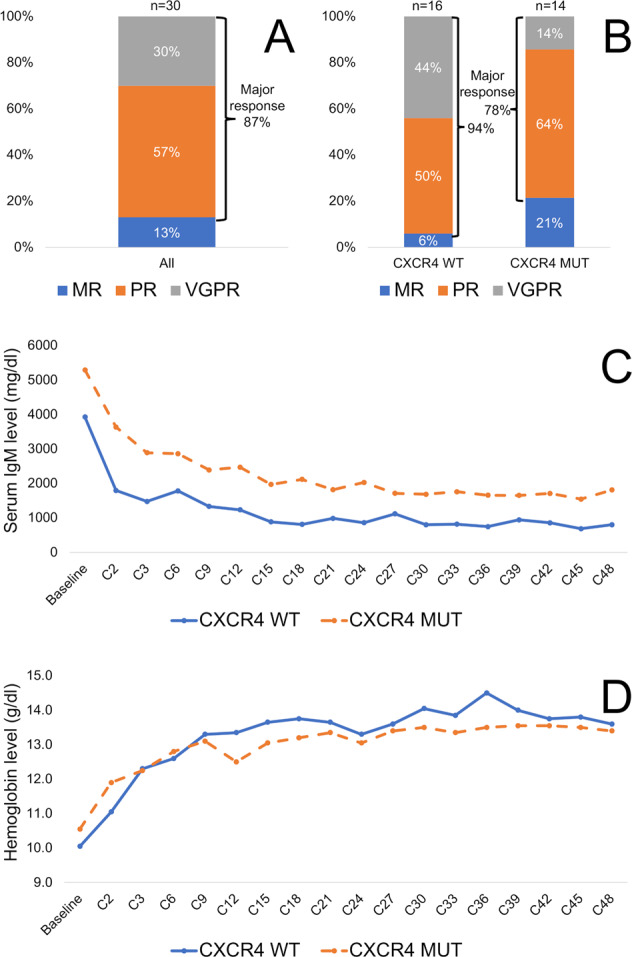
Response to therapy. Categorical responses at the best response in 30 treatment-naive patients with WM treated with ibrutinib for the entire cohort (**A**), and according to *CXCR4* mutational status (**B**); and serial serum IgM levels (**C**) and hemoglobin levels (**D**) throughout ibrutinib therapy according to *CXCR4* mutational status. MR minor response, PR partial response; VGPR very good partial response; WT wildtype; MUT mutated; C cycle.

At best response, nine patients (30%) attained a VGPR, 17 (57%) attained a PR, and 4 (13%) attained a minor response, for an ORR of 100% and a major response rate of 87% (Fig. 2A). No patient attained a CR. The ORR was 100% in patients with and without *CXCR4* mutations. Categorical responses according to *CXCR4* mutational status are shown in Fig. 2B. The one patient with a frameshift mutation attained a VGPR. Serial serum IgM and hemoglobin levels throughout ibrutinib therapy are shown in Fig. 2C and Fig. 2D, respectively. None of the clinical factors evaluated were associated with the attainment of a major response (Supplemental Table S1). There was a numerically, though not significant, lower VGPR rate in patients with than without *CXCR4* mutations (14% vs. 44%; OR 0.21, 95% CI 0.04–1.29; *p* = 0.09; Supplemental Table S2).

**Fig. 3:**
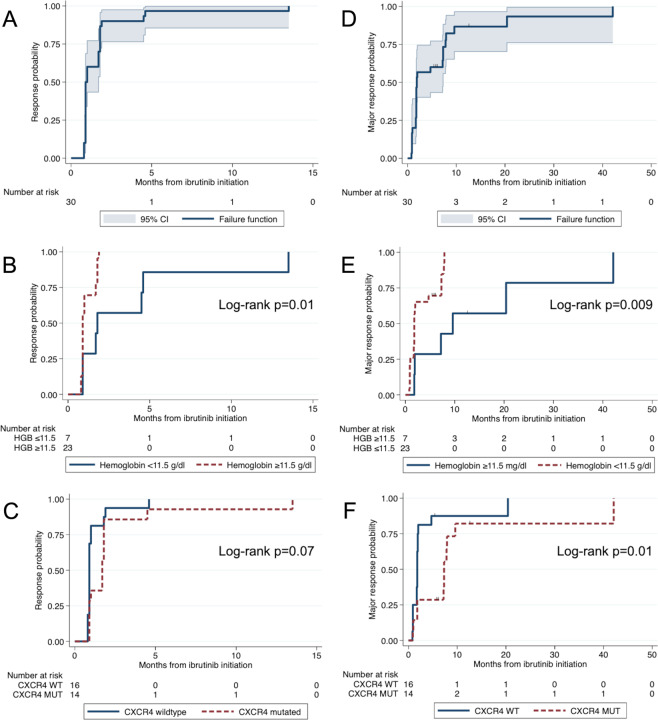
Time to response analyses. Time to response estimates in 30 treatment-naive WM patients treated with ibrutinib monotherapy for the entire cohort (**A**), and according to hemoglobin level (**B**) and CXCR4 mutational status (**C**), and time to major response estimates for the entire cohort (**D**), and according to hemoglobin level (**E**) and *CXCR4* mutational status (**F**). WT wildtype, MUT mutated.

The median time to a minor response was 0.9 months (95% CI 0.9–1.7; Fig. 3A). The median was longer in patients with hemoglobin level ≥11.5 g/dl than in patients with hemoglobin level <11.5 g/dl (1.8 months, 95% CI 0.9–4.6, and 0.9 months, 95% CI 0.9-1.0 months; *p* = 0.01; Fig. 3B). There was a trend toward longer median time to minor response in patients with *CXCR4* mutations than without *CXCR4* mutations (1.7 months, 95% CI 0.9–1.8, and 0.9 months, 95% CI 0.9–1.0; *p* = 0.07; Fig. 3C). The median time to major response was 1.9 months (95% CI 1.8–7.3; Fig. 3D). The median was longer for patients with hemoglobin level ≥11.5 mg/dl than for patients with hemoglobin level ≤11.5 mg/dl (9.6 months, 95% CI 1.8-not reached, and 1.8 months, 95% CI 1.7–4.7; *p* = 0.009; Fig. 3E) and for patients with *CXCR4* mutations than without *CXCR4* mutations (7.3 months, 95% CI 1.8–9.6, and 1.8 months, 95% CI 0.9–1.9; *p* = 0.01; Fig. 3F). *CXCR4* mutations (*p* = 0.02) and hemoglobin <11.5 g/dl (*p* = 0.02) were independently associated with a longer and a shorter time to major response, respectively, in a multivariate model including these two factors.

Of the ten patients with lymphadenopathy ≥1.5 cm, seven had a reduction, one had resolution, and two had stable lymphadenopathy on ibrutinib therapy for a lymph node response rate of 80%. Of the four patients with splenomegaly ≥15 cm, three experienced a reduction, and one experienced resolution on ibrutinib therapy for a spleen response rate of 100%. The three patients with aVWD experienced normalization of serum markers within six months, associated with a response to ibrutinib therapy. The patients with autoimmune hemolysis and cryoglobulinemia experienced cessation of symptoms within three months of ibrutinib therapy.

### Survival analysis

**Fig. 4:**
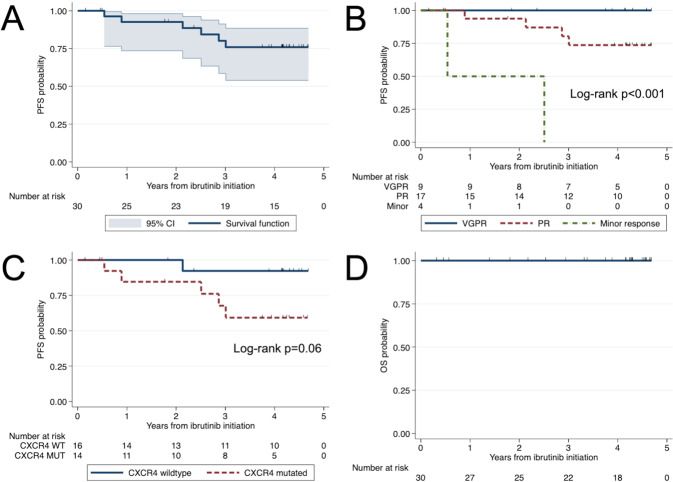
Survival analyses. Progression-free survival (PFS) estimates for the entire cohort (**A**), according to International Prognostic Scoring System for WM (**B**), and according to *CXCR4* mutational status (**C**), and overall survival (OS) estimates in 30 treatment-naive patients with WM treated with ibrutinib monotherapy. VGPR very good partial response, PR partial response, WT wildtype, MUT mutated.

At a median follow-up time of 50.1 months (95% CI 44.9–53.8), six patients have progressed. The median PFS was not reached (Fig. 4A). The 4-year PFS rate was 76% (95% CI 54–88%). The 4-year PFS rate was higher for patients who attained VGPR (100%, 95% CI not evaluable) than for patients who attained PR (74%, 95% CI 44–89%) or minor response (0%; 95% CI not evaluable) (*p* < 0.001; Fig. 4B). HR could not be calculated as none of the participants who attained a VGPR had progressed and all the participants who attained a minor response had progressed at 4 years. Five of the six patients (83%) who progressed had a *CXCR4* mutation. There was a non-significantly lower 4-year PFS rate in patients with than without *CXCR4* mutations (59%, 95% CI 28–81%, versus 92%, 95% CI 57–99%; *p* = 0.06; Fig. 4C). *CXCR4* mutations were associated with a sixfold increase in the risk of progression or death (HR 6.03, 95% CI 0.7–51.6; *p* = 0.09). No other evaluated factors were associated with a worse PFS (Supplemental Table S3). The one patient with a frameshift *CXCR4* mutation has not yet progressed at 55 months and continues on ibrutinib therapy.

There were no deaths while patients were on active therapy or follow-up, for an overall survival rate of 100% (Fig. 4D).

### Safety

Grade ≥2 treatment-related adverse events are shown in Table 2. Grade 4 events included ventricular fibrillation and thrombocytopenia (*n* = 1 each). The patient who experienced ventricular fibrillation was successfully resuscitated and taken off study. Grade ≥2 atrial fibrillation was reported in 20% of patients. The median time from ibrutinib initiation to atrial fibrillation diagnosis was 26 months (range 3–38). All patients were managed medically and continued ibrutinib therapy.

**Table 2:** Grade 2 or higher adverse events possibly, probably or definitely associated with ibrutinib therapy.

Adverse Event	Grade 2	Grade 3	Grade 4	Total Grades 2-4
Abdominal pain	2			2
Alanine aminotransferase increased		3		3
Anemia		2		2
Anorexia	1			1
Arthralgia	3			3
Aspartate aminotransferase increased		2		2
Atrial fibrillation	6			6
Bone pain	2			2
Cardiac arrest			1	1
Cough	4			4
Creatinine increased	3			3
Diarrhea	3			3
Drug-induced hepatitis		1		1
Edema	1			1
Fatigue	10			10
Flu-like symptoms	2			2
Headache	1			1
Hematoma	7	1		8
Hematuria	2			2
Hypertension	2	3		5
Lower tract respiratory infection	4	1		5
Mucositis, oral	3			3
Myalgia	3	1		4
Nausea	1			1
Neutropenia		3		3
Pleural effusion		1		1
Rash	3	2		5
Renal calculi	2			2
Sinus tachycardia	2			2
Soft tissue infection	4			4
Thrombocytopenia		1	1	2
Upper tract respiratory infection	9			9
Urinary tract infection	4	2		6
Urinary tract obstruction	1	1		2
Vasculitis	1			1
Ventricular fibrillation			1	1

Eight patients developed a malignancy while on active therapy or active follow-up. Four patients had a localized basal cell carcinoma of the skin (all treated with surgical excision), one had localized prostate cancer (treated with surgical resection), one had localized melanoma (treated with surgical excision), one had invasive rectal cancer (treated with surgery and radiotherapy), and one had esophageal cancer (taken off study to receive chemoradiotherapy). No patient transformed to diffuse large B-cell lymphoma or developed a myeloid neoplasm.

The ibrutinib dose was reduced in three patients. One patient was decreased to 280 mg/day after 36 months on therapy while in a VGPR due to rectal bleeding. One patient was decreased to 280 mg/day after 22 months on therapy while in a VGPR due to muscle cramps affecting activities of daily living, and one patient was decreased to 140 mg/day after 4 months of therapy while in a PR due to severe arthralgia. None of these patients had progressed at their last follow-up.

The median number of expected doses was 1360 (range 57–1430), the median number of doses held was 14 (range 0–54), and the median number of missed doses were 1 (0–23), for a median dose intensity of 99% (range 95–100%).

The median serum IgG level decreased from 562.5 mg/dl (range 191–3251 mg/dl) at baseline to 484 (range 159–1146 mg/dl) at the end of treatment (*p* = 0.03). The median serum IgA level decreased from 62 (range 11–576 mg/dl) at baseline to 51.5 (range 12–386 mg/dl) at the end of treatment (*p* = 0.42).

### Patient disposition

At the time of this report, all patients are off study therapy. Nineteen patients (66%) completed the 4-year study course of ibrutinib monotherapy and have transitioned to commercial ibrutinib. Eleven patients stopped study therapy before the 4-year mark because of disease progression (*n* = 4), ibrutinib-related adverse events (*n* = 2; ventricular fibrillation and liver injury), unrelated cardiac arrest as previously reported [7] (*n* = 1), unrelated esophageal cancer (*n* = 1), and travel constraints (*n* = 3). Two of the six patients with hematologic progression on ibrutinib continued therapy as they were deriving clinical benefit. Three patients started a new treatment; two patients started bendamustine and rituximab, and one patient started bortezomib, dexamethasone and rituximab. The one patient who had esophageal cancer did not receive additional WM-directed therapy. Of the three patients who were taken off study due to adverse events, one started bendamustine and rituximab, one started venetoclax, and one enrolled in a clinical trial.

Three patients died after they came off protocol therapy. One patient progressed after 36 months of therapy, was switched to bendamustine and rituximab, and died of septic shock 6 weeks after stopping ibrutinib. One patient was diagnosed with rectal cancer 3 months after completing study therapy and died 6 months later while on commercial ibrutinib. One patient was diagnosed with esophageal cancer after completing 45 months of ibrutinib therapy and died 2 months after stopping ibrutinib. These events were unrelated to ibrutinib treatment.

## Discussion

Herein, we present the final report from an investigator-initiated, prospective phase II study evaluating ibrutinib monotherapy in 30 treatment-naive patients with WM with a median follow-up time of 50 months. Our data show that ibrutinib monotherapy, as primary therapy for WM, induced a high rate of durable responses and further affirms ibrutinib as a standard of care in treatment-naive patients with WM.

Compared with our initial report of this study [7], the major response rate increased from 83% to 87% and the VGPR rate increased from 20% to 30%. Continued ibrutinib therapy was associated with deepening of response in patients with and without *CXCR4* mutations. The major response rate in patients without *CXCR4* mutations remained the same at 94%, though the major response rate in those with *CXCR4* mutations increased from 71% to 78%. The VGPR rate in patients without *CXCR4* mutations increased from 31% to 44%, and in those with *CXCR4* mutations from 7% to 14%. Similar findings were outlined in the final report from the pivotal study of ibrutinib in previously treated WM patients that supported regulatory approval of ibrutinib [12]. Furthermore, long-term follow-up from the INNOVATE main study and the Arm C sub-study also reported deepening of response with continued ibrutinib therapy [12, 13].

Rapid improvements in hemoglobin levels, many leading to complete normalization, were recognized in this study, independent of BM clearance and likely mediated by cytokine suppression [14]. Along with a serological response, there was a significant decrease in extramedullary disease, observed in over 80% of patients. Of interest was to observe the resolution of aVWD (all patients had *CXCR4* mutations) and autoimmune hemolytic anemia associated with response to ibrutinib. We had previously reported a high prevalence of low VWD markers in WM patients with nonsense *CXCR4* mutations [15].

Ibrutinib monotherapy in this frontline study produced durable responses. With a median follow-up time of 50 months, the median PFS was not reached. The 4-year PFS rate was 76%. This result compares favorably with the outcomes of other regimens used as primary therapy in patients with WM, including bendamustine and rituximab and bortezomib, dexamethasone, and rituximab, wherein median PFS of 4–6 years have been reported [16–18]. Moreover, the PFS rate with single-agent ibrutinib in treatment-naive patients was comparable with the INNOVATE study, wherein the 54-month PFS rate was 68% in the ibrutinib plus rituximab arm [19].

The outcomes observed in patients without *CXCR4* mutations were highly encouraging with a major response rate of 97%, a VGPR rate of 44%, and a 4-year PFS rate of 92%. Only one patient without a *CXCR4* mutation progressed to date. These results compare favorably with our previous experience in patients with relapsed or refractory WM where the major response rate was 97%, the VGPR rate was 47%, and the 5-year PFS rate was 70% [6]. These findings support that, based on the depth and durability of response observed, ibrutinib monotherapy should be considered the standard of care in WM patients who do not harbor *CXCR4* mutations.

*CXCR4* mutations were associated with numerically, though not significant, lower odds of attaining VGPR and a higher risk of progression in our patients, possible because of the small sample size. This result is consistent with our previous experience with ibrutinib monotherapy in patients with relapsed and/or refractory WM [12]. Patients with *CXCR4* mutations had a median PFS of 4 years. Over 40 different *CXCR4* mutations have been reported in patients with WM, which can be subdivided in nonsense and frameshift mutations [1, 20]. Preclinically, *CXCR4* mutations were associated with resistance to BTK inhibitors mediated by overexpression of ERK and AKT [4]. In a retrospective study of 180 patients with WM, we observed fewer major responses and shorter PFS with ibrutinib monotherapy in patients with nonsense *CXCR4* mutations [21]. Patients with frameshift *CXCR4* mutations had similar response and PFS outcomes to patients without *CXCR4* mutations. In the present study, only one patient had a frameshift *CXCR4* mutation and, therefore, we could not determine differences in outcomes between patients with nonsense and frameshift mutations. Nonetheless, ibrutinib monotherapy induced durable responses in WM patients with *CXCR4* nonsense mutations. A phase I study evaluating the combination of ibrutinib and the anti-CXCR4 antibody ulocuplumab was associated with rapid and deep responses in patients with WM harboring *CXCR4* mutations (manuscript submitted). A prospective phase Ib study evaluating the combination of ibrutinib and the CXCR4 inhibitor mavorixafor in this patient population is ongoing (NCT04274738).

The adverse event profile of ibrutinib in this study showed no unexpected toxicities. All patients who developed atrial fibrillation were managed medically and continued ibrutinib therapy, emphasizing that atrial fibrillation is not a contraindication for or a reason to discontinue ibrutinib therapy as long as the patient is benefiting from therapy. The novel BTK inhibitor zanubrutinib has been associated with lower rates of atrial fibrillation than ibrutinib in a randomized study and could be considered in patients at a high risk of atrial fibrillation [22]. The risk of ventricular arrhythmias on ibrutinib is low and should be discussed with the prospective patient before initiating therapy [23, 24]. Dose reductions were necessary in 5% of patients. However, all these patients were in a major response at the time of the dose reduction. The median dose intensity was 99% in this study, suggestive of a high degree of compliance with ibrutinib therapy.

One of the limitations of the present study is the small sample size. However, our cohort is representative of the population of treatment-naive patients with WM based on age, sex, and other laboratory parameters at baseline. In addition, *CXCR4* testing was performed in a research setting and might not be replicated elsewhere.

In summary, our findings show that ibrutinib monotherapy is highly active with long-term disease control in treatment-naive patients with WM. While ibrutinib responses were affected by *CXCR4* mutations, long-term disease control was attained regardless of *CXCR4* mutational status. Furthermore, treatment was well tolerated, with no unexpected toxicities. The findings further establish ibrutinib as one of the most active agents in WM. Prospective, randomized studies against other commonly used treatment options, such as bendamustine and rituximab, other BTK inhibitors, and combinations with CXCR4 or BCL2 inhibitors, are needed to further define the optimal use of ibrutinib in patients with WM.

## Supplementary information

Supplemental data
