# Supplementary material for: Long-term follow-up of ibrutinib monotherapy in treatment-naive patients with Waldenstrom macroglobulinemia
Source: Leukemia. 2021 Sep 16;36(2):532–9. doi: 10.1038/s41375-021-01417-9 (PMC8807393; doi:10.1038/s41375-021-01417-9)
Supplement: Supplementary file 1 — Supplemental data [file 41375_2021_1417_MOESM1_ESM.docx]

**Supplemental data**

**Table S1.** Logistic regression analyses for major response to ibrutinib

| **Variable** | **OR (95% CI)** | **p-value** |
| --- | --- | --- |
| Age ≥65 years | 0.53 (0.05-5.86) | 0.61 |
| Male sex | UTC* |  |
| Serum IgM ≥4,000 mg/dl | 4.10 (0.37-44.8) | 0.25 |
| Hemoglobin ≤11.5 g/dl | 1.11 (0.10-12.8) | 0.93 |
| Platelet count <100 K/uL | 0.12 (0.01-2.46) | 0.17 |
| Beta-2-microglobulin >3.5 mg/l | 0.90 (0.08-10.2) | 0.94 |
| Bone marrow involvement ≥60% | 0.90 (0.08-10.2) | 0.94 |
| IPSSWM |  |  |
| Low risk | 1.00 (reference) |  |
| Intermediate risk | 0.75 (0.09-6.39) | 0.79 |
| High risk | UTC** |  |
| *CXCR4* mutation | 0.24 (0.02-2.68) | 0.25 |
| Time from WM to ibrutinib <12 months | 0.24 (0.02-2.68) | 0.25 |

UTC: unable to calculate

*All female participants attained a major response

**The high-risk group was omitted because of collinearity

**Table S2.** Logistic regression analyses for very good partial response to ibrutinib

| **Variable** | **OR (95% CI)** | **p-value** |
| --- | --- | --- |
| Age ≥65 years | 0.63 (0.13-3.09) | 0.56 |
| Male sex | 1.09 (0.17-7.06) | 0.93 |
| Serum IgM ≥4,000 mg/dl | 0.34 (0.02-1.88) | 0.14 |
| Hemoglobin ≤11.5 g/dl | 3.20 (0.32-31.4) | 0.32 |
| Platelet count <100 K/uL | UTC* |  |
| Beta-2-microglobulin >3.5 mg/l | 4.00 (0.41-38.7) | 0.23 |
| Bone marrow involvement ≥60% | 1.40 (0.22-8.77) | 0.72 |
| IPSSWM |  |  |
| Low risk | 1.00 (reference) |  |
| Intermediate risk | 1.50 (0.12-19.4) | 0.76 |
| High risk | 2.22 (0.19-25.7) | 0.52 |
| *CXCR4* mutation | 0.21 (0.04-1.29) | 0.09 |
| Time from WM to ibrutinib <12 months | 0.44 (0.10-2.48) | 0.29 |

UTC: unable to calculate

*None of the participants with platelet count <100 K/uL attained a VGPR

**Table S3.** Cox proportional-hazard regression analyses for progression-free survival on ibrutinib

| **Variable** | **HR (95% CI)** | **p-value** |
| --- | --- | --- |
| Age ≥65 years | 0.28 (0.05-1.51) | 0.14 |
| Male sex | 0.25 (0.05-1.36) | 0.12 |
| Serum IgM ≥4,000 mg/dl | 1.91 (0.35-10.4) | 0.46 |
| Hemoglobin ≤11.5 g/dl | 1.80 (0.21-15.4) | 0.59 |
| Platelet count <100 K/uL | UTC* |  |
| Beta-2-microglobulin >3.5 mg/l | 0.36 (007-1.77) | 0.21 |
| Bone marrow involvement ≥60% | 0.79 (0.15-4.35) | 0.79 |
| IPSSWM |  |  |
| Low risk | 1.00 (reference) |  |
| Intermediate risk | 0.69 (0.11-4.12) | 0.68 |
| High risk | 0.18 (0.02-1.96) | 0.16 |
| *CXCR4* mutation | 6.03 (0.70-51.6) | 0.09 |
| Time from WM to ibrutinib <12 months | 1.32 (0.25-6.11) | 0.80 |

UTC: unable to calculate

*None of the participants with platelet count <100 K/uL have progressed yet
